# Redefining Surgical Collaboration: The First Structured Transcontinental Robotic Telementoring Experience Between Africa and South America

**DOI:** 10.1590/S1677-5538.IBJU.2026.0136

**Published:** 2026-03-03

**Authors:** Marcio Covas Moschovas, Renan Desimon Cabral, Eduardo Parra-Davila, Vanessa Alvarenga-Bezerra, Antônio Rebello Horta Gorgen, Tiago Almeida Ramos, Pablo Wesz Nascimento, Guilherme Arend Pesce, Herber Sauer, Mirandolino Batista Mariano, Vipul Patel

**Affiliations:** 1 AdventHealth Global Robotics Institute Kissimmee FL United States AdventHealth Global Robotics Institute, Kissimmee, FL, United States; 2 University of Central Florida - UCF Orlando FL United States University of Central Florida - UCF, Orlando, FL, United States; 3 Hospital Mãe de Deus Porto Alegre RS Brasil Hospital Mãe de Deus, Porto Alegre, RS, Brasil; 4 Good Samaritan Medical Center West Palm Beach FL United States Good Samaritan Medical Center, West Palm Beach, FL, United States; 5 Hospital Israelita Albert Einstein São Paulo SP Brasil Hospital Israelita Albert Einstein, São Paulo, SP. Brasil

## COMMENT

Robotic surgery has progressively transformed minimally invasive surgery by expanding technical precision, ergonomics, and visualization (1). More recently, telementoring and telesurgery-enabled platforms have emerged as potential strategies to mitigate geographic disparities in access to specialized surgical expertise (2–4). In this context, the landmark transcontinental robotic surgery performed between Orlando (United States) and Luanda (Angola) demonstrated the technical feasibility and clinical safety of long-distance surgical collaboration and served as a foundational proof of concept for the current telesurgery scenario worldwide (5). Similarly, the present study was designed to expand the scope of telesurgery-enabled care and to evaluate whether a similar, ethically structured model could be translated to other regions of the globe, particularly within the American continent. In this study we report expert insights from the first transcontinental telesurgery study performed in Brazil, using a structured telementoring framework during robot-assisted radical prostatectomy.

In this experience, the patient and operative team were located in Brazil, while the remote surgeon was physically based in Angola. To specifically evaluate feasibility and safety, the procedure was performed by an experienced local robotic urology team at Hospital Mãe de Deus (Porto Alegre, Brazil), with the remote surgeon at Hospital Cardeal Dom Alexandre do Nascimento (Luanda, Angola) participating exclusively in a telementoring role. The interaction consisted of real-time intraoperative guidance, technical discussion, and exchange of surgical strategies. Full procedural responsibility, including initiation, execution of critical steps, and completion of the surgery, remained entirely with the local operating surgeon, thereby preserving local surgical autonomy while safely integrating international expertise into the operative workflow.

The study was conducted under full ethical and regulatory oversight, with approval by the Comitê de Ética em Pesquisa (CEP) of Hospital Mãe de Deus and formal registration on Plataforma Brasil under the Universal Trial Number (UTN): U1111-1331-3810. All procedures were performed in accordance with the Declaration of Helsinki and national regulations governing research involving human subjects. The explicit inclusion of institutional review, national registration, and transparent documentation was central to ensuring regulatory legitimacy and reproducibility.

A transcontinental connection was established between Luanda and Porto Alegre through a multi-segment public fiber-optic network, comprising a submarine fiber route from Luanda to Fortaleza (Brazil), followed by a terrestrial fiber from Fortaleza to Porto Alegre. The estimated end-to-end fiber distance was approximately 9,000 km. Biomedical and network engineers were present at both endpoints throughout the procedure, continuously monitoring latency, jitter, frame loss, and overall link stability, allowing real-time quality assessment and immediate response to any connectivity degradation during the telementoring phase.

A crucial component of this project was the use of a dedicated, procedure-specific informed consent for Telesurgery, developed based on prior FDA-approved experience between Orlando and Luanda (5). This consent addressed the role of the remote surgeon, cybersecurity safeguards, predefined connectivity thresholds (including latency and jitter limits), intraoperative contingency protocols, data protection measures, and the patient's right to withdraw consent at any time. The separation of telesurgery consent from standard surgical consent represents a relevant ethical advance in telesurgery-enabled care. Both teams followed a predefined contingency protocol, which was explicitly discussed with the patient as part of the consent process (6, 7).

During the Telesurgery, surgeons at both ends of the connection engaged in real-time discussion of surgical techniques and exchanged robotic console control at predefined key steps of the procedure. The local surgeon in Porto Alegre maintained full control and ultimate responsibility throughout the operation. At selected moments, the remote surgeon temporarily assumed console control to demonstrate technical variations and highlight relevant anatomical landmarks. Supplementary Video 1 illustrates the key operative steps and the knowledge exchange that occurred among both teams during the procedure. This illustrates the potential of Telesurgery for surgical education and telementoring, while minimizing complications.

The case involved a 62-year-old male patient diagnosed with acinar prostate adenocarcinoma, ISUP Grade Group 2 (Gleason score 3+4=7), with a preoperative PSA of 6.47 ng/mL and normal Digital rectal exam (DRE). A robot-assisted radical prostatectomy was performed on November 5th, 2025, using the MicroPort Toumai robotic platform. Total operative time was 90 minutes, with no intraoperative or postoperative complications. During active participation of the remote surgeon, mean network latency was 132 ms (range 129–137 ms), with 0% frame loss and no detectable jitter, indicating stable and reliable connectivity throughout the telementoring phase. Final pathology demonstrated Gleason 3+4, organ-confined disease involving both lobes, with approximately 10% tumor volume, negative surgical margins, no capsular invasion. At 45 days of follow-up, the patient reported undetectable PSA levels (<0.008 ng/mL), complete urinary continence, and full recovery of erectile function without the use of phosphodiesterase-5 inhibitors.

Beyond this individual urologic procedure, the project was conceived as a multispecialty study involving urology, gynecology, and general surgery. Although only the urologic case has been completed to date, subsequent procedures in gynecology and general surgery are planned under the same ethical, technical, and regulatory framework, highlighting the scalability of structured telementoring beyond a single specialty.

The innovation of this project lies not only in its geographic distance, but also in its concept. To our knowledge, this represents the first clinical telesurgery-related telementoring study performed in Brazil and the first live surgical collaboration between Africa and South America (7). More importantly, it demonstrates that telementoring can be safely implemented within a robust ethical framework that prioritizes patient autonomy, transparency, and institutional accountability. Rather than replacing local expertise or increasing predatory competition between remote experts and local surgeons, this approach promotes bidirectional learning and collaborative refinement of surgical practice, with potential to enhance robotic surgery training worldwide.

In summary, this telementoring experience during robot-assisted radical prostatectomy suggests that structured Telesurgery can be performed safely across Brazil and Africa. However, we still need more data under IRB-approved studies to establish these benefits in clinical settings and other specialties. In this context, although technology is innovative, it comes with clear responsibilities, including strict ethical oversight, reliable connectivity, and transparent patient consent. When these elements are in place, telementoring can improve surgical training, facilitate real-time exchange of expertise, and connect multiple centers simultaneously. This approach may help expand access to specialized knowledge while maintaining patient safety and local surgical responsibility worldwide.
